# The CD4^+^AT2R^+^ T cell subpopulation improves post-infarction remodelling and restores cardiac function

**DOI:** 10.1111/jcmm.12574

**Published:** 2015-05-20

**Authors:** Anna Skorska, Stephan von Haehling, Marion Ludwig, Cornelia A Lux, Ralf Gaebel, Gabriela Kleiner, Christian Klopsch, Jun Dong, Caterina Curato, Wassim Altarche-Xifró, Svetlana Slavic, Thomas Unger, Gustav Steinhoff, Jun Li, Robert David

**Affiliations:** a Reference and Translation Centre for Cardiac Stem Cell Therapy (RTC)/Department of Cardiac Surgery, University of RostockRostock, Germany; b Center for Cardiovascular Research and Department of Cardiology, Campus Virchow-Klinikum, Charité – Universitätsmedizin BerlinBerlin, Germany; c University of Göttingen Medical SchoolGöttingen, Germany; d German Rheumatism Research CentreBerlin, Germany; e Center for Cardiovascular Research (CCR) and Institute of Pharmacology, Charité - Universitätsmedizin BerlinBerlin, Germany; f Clinical Stem Cell Research Center and Department of Cardiovascular Surgery, Renji Hospital, School of Medicine, Shanghai Jiao Tong UniversityShanghai, China

**Keywords:** angiotensin II type 2 receptor, renin angiotensin system, CD4^+^ lymphocytes, myocardial infarction, heart failure cardiac remodelling

## Abstract

Myocardial infarction (MI) is a major condition causing heart failure (HF). After MI, the renin angiotensin system (RAS) and its signalling octapeptide angiotensin II (Ang II) interferes with cardiac injury/repair *via* the AT1 and AT2 receptors (AT1R, AT2R). Our study aimed at deciphering the mechanisms underlying the link between RAS and cellular components of the immune response relying on a rodent model of HF as well as HF patients. Flow cytometric analyses showed an increase in the expression of CD4^+^ AT2R^+^ cells in the rat heart and spleen post-infarction, but a reduction in the peripheral blood. The latter was also observed in HF patients. The frequency of rat CD4^+^ AT2R^+^ T cells in circulating blood, post-infarcted heart and spleen represented 3.8 ± 0.4%, 23.2 ± 2.7% and 22.6 ± 2.6% of the CD4^+^ cells. CD4^+^ AT2R^+^ T cells within blood CD4^+^ T cells were reduced from 2.6 ± 0.2% in healthy controls to 1.7 ± 0.4% in patients. Moreover, we characterized CD4^+^ AT2R^+^ T cells which expressed regulatory FoxP3, secreted interleukin-10 and other inflammatory-related cytokines. Furthermore, intramyocardial injection of MI-induced splenic CD4^+^ AT2R^+^ T cells into recipient rats with MI led to reduced infarct size and improved cardiac performance. We defined CD4^+^ AT2R^+^ cells as a T cell subset improving heart function post-MI corresponding with reduced infarction size in a rat MI-model. Our results indicate CD4^+^ AT2R^+^ cells as a promising population for regenerative therapy, *via* myocardial transplantation, pharmacological AT2R activation or a combination thereof.

## Introduction

Cardiac diseases are the leading causes of mortality and morbidity in industrialized countries. Despite many improvements in prevention, diagnosis and management, prevalence of heart failure (HF) is still rising, and small progress has been achieved in terms of survival prolongation 1.

Myocardial infarction (MI) is the major condition leading to HF. In experimental animal models, MI is commonly induced by induction of the left anterior descending coronary artery (LAD) 2. The resulting inflammatory response comprises attenuated activation of cytokines and adhesion molecules, ventricular dilatation and interstitial fibrosis in the non-infarcted myocardium. This remodelling process leads to changes in the left ventricle (LV) geometry, which contribute to the development of HF 3–5.

Following MI, neurohumoral activation involving the renin angiotensin system (RAS) leads to persistent vasoconstriction, LV hypertrophy, sympathetic nervous activation and endothelial dysfunction 6,7. Suppression of RAS may become an important means for treatment of HF after MI. Angiotensin II (AngII), a RAS octapeptide found at higher concentrations in the heart tissue than in plasma 8, acts through two known receptors: angiotensin II type 1 receptor (AT1R) and angiotensin II type 2 receptor (AT2R) 9. The mere 32–34% sequence homology between both receptors reflects their different functions. AT1R is mostly responsible for sodium and water retention, vasoconstriction, cellular growth, proliferation and endothelial dysfunction 10. To date, AT2R has been reported to exert anti-inflammatory 11,12, anti-apoptotic, anti-fibrotic 13,14 and protective 15 functions. Furthermore, AngII induces interactions between leucocyte and endothelial niches *via* AT1- and AT2R 16. Moreover, an up-regulated level of AT2R during ischemic cardiovascular injury 17,18 speaks in favour of its potential role in regulating adaptive cardiovascular repair.

Guzik *et al*.^19^ have shown that upon long-term infusion of AngII in mice, the number of peripheral blood T lymphocytes was increased. Kvakan *et al*. 20 observed a beneficial role of naturally occurring T regulatory cells after adoptive transfer into AngII-induced inflamed mice exhibiting cardiac damage. Additionally, the preventive role of post-MI activated AT2R-expressing CD8^+^ T cells secreting interleukin-10 (IL-10) has been reported by our group with the exception that only post-MI activated AT2R-expressing CD8^+^ T cells, which secreted IL-10 reduced the infarction size in MI recipient rats 18. This cell population reduced the infarction size in MI recipient rats.

To clarify the inflammatory mechanisms following MI and the activation of RAS, we analysed the expression of AT2R on CD4^+^ cells in human and in a rat model. In this study, CD4^+^ AT2R^+^ were shown for the first time to act as a ‘regulatory’ T cell subset facilitating cardiac regeneration, as evident from improved cardiac function and reduced infarction size.

## Materials and methods

### Rat model of myocardial infarction

Myocardial infarction was induced in adult male Wistar rats (200–220 g) as described previously 17,18. Briefly, rats were anaesthetized with ketamin/xylazine (Sigma-Aldrich, Seelze/Hannover, Germany) 80 mg/10 mg/kg i.p., incubated and ventilated with a small animal ventilator (Harvard Apparatus,March-Hugstetten, Germany). After thoracotomy, a suture was placed around the proximal LAD coronary artery. Sham-operated rats underwent the same surgical procedure without coronary ligature. Rats were killed at 1 or 4 weeks after operation. Animal housing, care and applications of experimental procedures complied with the German law on animal protection.

### Immunofluorescence staining

Immunofluorescence staining was performed according to standard protocols. For details please refer to Data S1.

### Isolation and flow cytometry analysis of CD4^+^ AT2R^+^ T cells from heart, spleen and blood

CD4^+^ T cells were isolated from heart, spleen and blood samples at 1 or 4 weeks after MI or sham-operation as described previously 17,18, with slight modifications. Briefly, cardiac mononuclear cells were obtained following digestion and density gradient sedimentation. Mononuclear cell suspensions prepared from heart, spleen and blood samples were stained with mouse anti-CD4-phycoerythrin (PE) (1:10; eBioscience, Vienna, Austria) and polyclonal anti-AT2 (1:50; Santa Cruz Biotechnology, Dallas, Texas, USA), followed by anti-PE Microbeads (1:10, Miltenyi Biotec GmbH, Bergisch Gladbach, Germany) and donkey anti-rabbit Alexa Fluor 488 (1:50; Life Technologies GmbH, Darmstadt, Germany). CD4^+^ T cells were enriched using magnetic activated cell sorting (MACS; Miltenyi Biotec). CD4^+^ T cell populations were analysed using flow cytometry on a FACS LSRII® or, sorted (CD4^+^ AT2R^+^ and CD4^+^ AT2R^−^ cells) on FACSAria® (BD Bioscience, Heidelberg, Germany) using BD FACS Diva software (version 6.1.2; BD Bioscience).

### qRT-PCR

qRT-PCR was performed according to standard protocols. For details please refer to Data S1.

### *Ex vivo* characterization of CD4^+^ AT2R^+^ T cells

Blood mononuclear cells were stained with primary antibodies [rabbit anti-AT2R or goat anti-AT2R polyclonal (Santa Cruz Biotechnology, each 1:50)], then secondary antibodies [donkey anti-rabbit Alexa 488 (1:50) or donkey anti-goat allophycocyanin (APC; 1:40; R&D Systems, Wiesbaden-Nordenstadt, Germany)], mouse anti-CD4-PE (1:40; eBioscience) or mouse anti-CD4-FITC (1:40; eBioscience)., Intracellular staining was performed with mouse anti-FoxP3-APC (1:40; BD Bioscience), mouse anti-FoxP3-PE (1:50; BD Bioscience), mouse anti-CD25-APC (1:40; BD Bioscience), rat anti-IL-10-APC (1:50; BD Bioscience) or mouse anti-tumour necrosis factor (TNF)-α-PE-Cy7 (1:40; eBisocience). At least 1 × 10^4^ events in the CD4^+^ cells gate were acquired for each sample.

### Functional role of AT2R in cytokine expression of CD4^+^ AT2R^+^ T cells

To investigate an effect of AT2R stimulation on cytokine expression, freshly sorted human blood CD4^+^ AT2R^+^ and CD4^+^ AT2R^−^ T cells were cultured in U-bottom 96-well plates at a density of 10^6^ cells/ml in RPMI 1640 medium supplemented with 10% FBS (Fetal Bovine Serum, PAN-Biotech, Aidenbach, Germany). Cultured cells were exposed to Ang II (0.5 nM; Sigma-Aldrich) in the presence or absence of AT2R blocker PD123319 (PD; 5 nM; Tocris Bioscience, Bristol, United Kingdom). After 24 hrs, cells were harvested for intracellular cytokine staining of IL-10/TNF-α and flow cytometric analysis.

### Preparation of donor CD4^+^ AT2R^+^ T cells and intramyocardial transplantation

Donor CD4^+^ AT2R^+^ and CD4^+^ AT2R^−^ T cells were prepared from spleens of male rats 7 days after induction of MI. Immediately after LAD ligation, 2.5 × 10^5^ CD4^+^ AT2R^+^ or CD4^+^ AT2R^−^ T cells resuspended in 50 μl saline were injected into the border zone of the ischemic myocardium of each recipient female rat. Myocardial infarction rats injected with saline served as control group.

### Evaluation of cardiac injury

Four weeks after MI and cell transplantation, recipient rats were killed. Cardiac injury was **analyzed** as described previously 21. Briefly, heart sections of four horizontal infarct levels (5 μm) were stained with Fast Green FCF (Sigma-Aldrich) and Sirius Red (Division Chroma). Stained sections were mounted with FluorSave™ Reagent (Merck Chemicals Ltd., Darmstadt, Germany), and visualized under Leica DMLB fluorescence microscope equipped with a digital camera (type DFC 420C; Leica Camera AG, Wetzlar, Germany). Sirius Red positive stained areas in the remote area near endocardial border were acquired in ten randomly chosen fields per section (two sections/level) with Leica Application Suite software (LAS, version 2.7.1 R1) using 40× Plan-Achromat objective. Two contiguous levels of the heart which represent the major infarct ratio were **analyzed** using computerized planimetry (Axio Vision LE Rel. 4.5 software; Carl Zeiss GmbH, Jena, Germany). The ratio of scar length and entire circumference defined the infarct extent for the endocardial and epicardial surfaces, respectively, The infarct area was determined as the average of endocardial and epicardial surfaces and was given in percent.

### Evaluation of cardiac function

Four weeks after MI and cell transplantation, recipient rats were subjected to pressure-volume (P/V) loop measurements using the Millar Pressure-Volume System (Catheter model SPR-838), Millar Pressure Conductance Unit (model MPCU-200) and PowerLab data acquisition hardware (emka Technologies, Paris, France). Following a small incision in the external jugular vein, a plastic catheter was inserted and 200 UI/kg of heparin was administrated. Up to 0.4 ml blood was collected, immediately applied into two cylindrical holes with defined volumes (95 or 300 μl) and calibration of volume was performed. Thereafter, calibration of pressure at 0 and 100 mmHg was performed. The right carotid artery was subsequently exposed by a small incision on the neck and the laterally retraction of the osmohyoid muscles. After a small incision between the two ligatures, the Millar catheter was carefully inserted. To secure the catheter, the loose ligature around it was tied. Values of parallel conductance volume (Vp) were acquired *via* thrice injection of PBS, averaged and used for the correction of conductance volume. IOX Version 1.8.3.20 software (emka Technologies) was used to analyse all P/V loop data recorded at steady-state (baseline) and under stress conditions (Dobutamine, 10 μg/kg/min. i.v., Sigma-Aldrich).

### Detection of cell engraftment by fluorescent *in situ* hybridization

Fluorescent in situ hybridization (FISH) was performed according to standard protocols. For details please refer to ‘Data S1’.

### Isolation and analysis of human circulating CD4^+^ AT2R^+^ T cells

Peripheral venous blood was obtained from patients with a clinical diagnosis of chronic HF according to current guidelines of the European Society of Cardiology and American Heart Association at the Department of Cardiology, Campus Virchow – Klinikum. Blood from healthy donors was obtained from the Institute for Transfusion Medicine, Charité-Universitätsmedizin Berlin. Written informed consent according to the Declaration of Helsinki was received prior to inclusion in the study. Around 25 ml of blood was drawn from an antecubital vein into K-ethylenediaminetetraacetic acid tubes and processed within 8 hrs. Mononuclear cells were isolated by density gradient centrifugation at 400 × g at 20°C for 35 min. The CD4^+^ T cells were isolated by MACS as described above. For cytokine measurement, enriched CD4^+^ T cells were further divided into CD4^+^ AT2R^+^ and CD4^+^ AT2R^−^ T cells using fluorescence-activated cell sorting. Alternatively, enriched CD4^+^ T cells were also stained with primary rat anti-human/mouse CD44-eFluor 450 and mouse anti-human CD62L-PE-Cy7 (each 1:40; eBioscience) and directly subjected to FACS analysis.

### Statistics

Results were expressed as mean ± SEM. Two-group comparisons were analysed by two-tailed Student’s *t*-test. Multiple comparisons were analysed with one-way anova followed by Bonferroni *post hoc* test. Differences were considered significant at a value of *P* < 0.05.

## Results

### Human CD4^+^AT2R+ T cell population in health and heart failure

**Figure 1:**
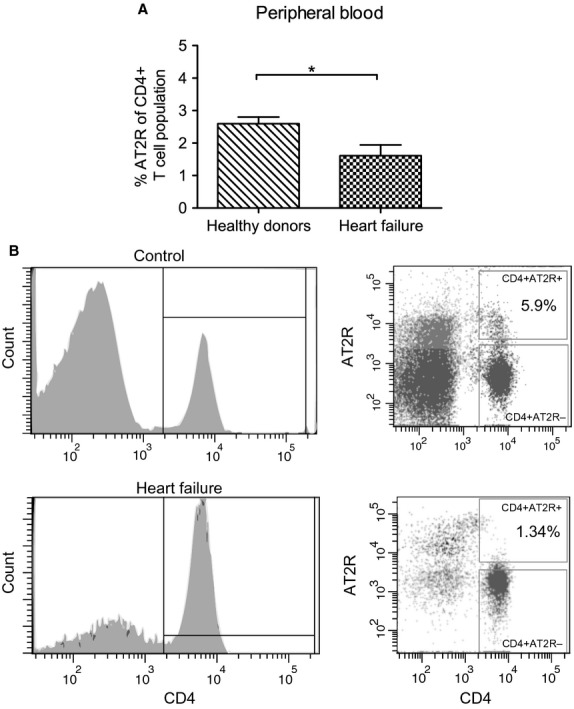
CD4^+^AT2R^+^ T cells were reduced in HF patients (A) *P < 0.05 (heart failure, *n* = 9, healthy donors, *n* = 27). (B) Representative FACS plots from healthy controls (upper panel) and HF patients (lower panel).

**Figure 2:**
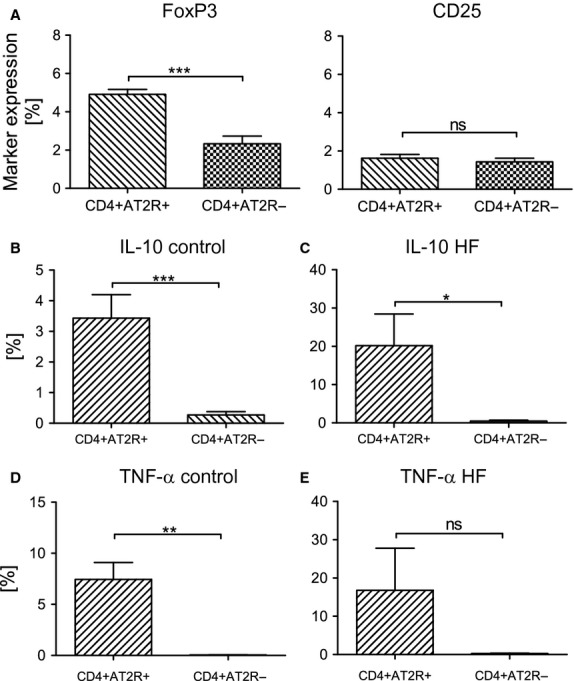
Characterization of human blood CD4^+^ AT2R^+^ T cells. Flow cytometric analysis was performed on gated CD4^+^ AT2R^+^ and CD4^+^ AT2R^−^ subpopulations of mononuclear cells (MNCs) from healthy donors (A, Foxp3, *n* = 8; CD25, *n* = 11); Predominant IL-10 expression was observed in the CD4^+^AT2R^+^ (*versus* CD4^+^ AT2R^−^) T cells of healthy controls (B, *n* = 6) and HF patients (C, *n* = 7). Increased TNF-α expression was observed in the CD4^+^ AT2R^+^ (*versus* CD4^+^ AT2R^−^) T cells of healthy controls (D, *n* = 6) but not HF patients (E, *n* = 7). ns, not significant; **P < 0.01, ***P < 0.001.

The baseline characteristics of patients with HF are presented in Table S1. A CD4^+^ AT2R^+^ T cell population was detected by flow cytometry in mononuclear cells isolated from peripheral blood of both patients with HF (Fig. S1A) and healthy controls. AT2R expression was confirmed on mRNA level (Fig. S1B). To study the adaptive distribution of CD4^+^ AT2R^+^ T cells during cardiac injury, we compared the frequency of these cells in peripheral blood of patients with HF with those of healthy controls. The frequency of CD4^+^ AT2R^+^ T cells in blood CD4^+^ T cells was reduced from 2.6 ± 0.2% in healthy controls to 1.7 ± 0.4% in patients with HF (Fig.1A), revealing a potential accumulation of CD4^+^ AT2R^+^ T cells infiltrating the failing heart. As a result of the essential role of regulatory T cells (Treg) in controlling immune response under physiological and pathological conditions, we have addressed the expression of immunoregulatory transcription factor FoxP3, Treg surface marker CD25 within the CD4^+^ AT2R^+^ T cell subset, and the potential to produce anti-inflammatory IL-10 cytokine. In human CD4^+^ AT2R^+^ T cells, the frequency of FoxP3-positive cells was increased by 2.1-fold (Fig.2, *P* < 0.0001), the frequency of IL-10-secreting cells was increased by 12.6-fold (healthy donors) and 41.2-fold (HF) compared to CD4^+^ AT2R^−^ T cells (*P* < 0.01, *P* < 0.05, respectively; Fig.2B and C). However, no difference in CD25 expression was detected (Fig.2, right plot).

**Figure 3:**
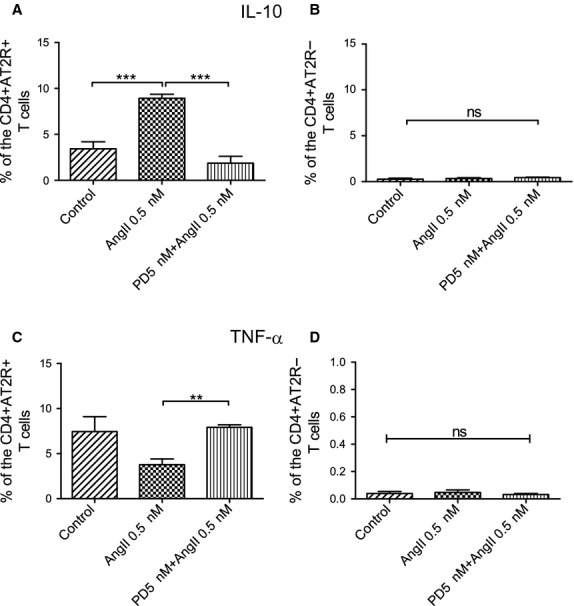
IL-10 and TNF-α expression in human blood CD4^+^ AT2R^+^ T cells. Sorted cells were analysed after 1 day of cultivation. AT2R mediated IL-10 production in the CD4^+^ AT2R^+^ (A) but not in the CD4^+^ AT2R^−^ (B) T cells of healthy controls as observed after AT2R stimulation with angiotensin II (AngII) in presence or absence of AT2R blocker PD123319 (PD). Increased TNF-α expression was observed in the CD4^+^ AT2R^+^ (*versus*CD4^+^ AT2R^−^) T cells of healthy controls (C, *n* = 6) but not HF patients (B, *n* = 7). AT2R shows a tendency to down-regulate TNF-α production in the CD4^+^ AT2R^+^ (C) but not in CD4^+^ AT2R^−^ (D) T cells of healthy donors, as shown after AT2R stimulation with AngII in the presence or absence of AT2R blocker PD123319 (PD). ns, not significant; **P < 0.01.

To address a potential cardioprotective role of AT2R, we determined whether AT2R influenced the regulation of inflammatory-related cytokines IL-10 and TNF-α in the CD4^+^ AT2R^+^ T cells. Upon AngII stimulation, IL-10 expression in the CD4^+^ AT2R^+^, but not in CD4^+^ AT2R^−^ T cells was significantly increased (2.6-fold in comparison to the control group, Fig.3A and B), while TNF-α expression in the CD4^+^ AT2R^+^ T cells was reduced 2.0-fold (Fig.3C and D). Both effects were completely abolished by the AT2R blocker PD123319. Interestingly, a highly increased level of TNF-α expression was observed exclusively in the CD4^+^ AT2R^+^ T cells (Fig.2D and E).

**Figure 4:**
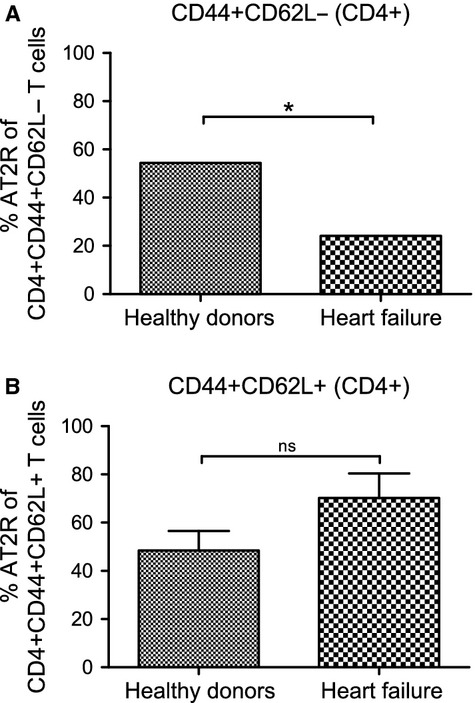
Distribution of the CD4^+^ AT2R^+^ T cells within CD4^+^ CD44^+^ CD62L^−^ or CD4^+^ CD44^+^ CD62L^+^ T cell subsets A. A decreased frequency of CD4^+^ AT2R^+^ T cells within the CD4^+^ CD44^+^ CD62L^−^ cell subset was observed in patients with HF. No significant difference was detected within the CD4^+^ CD44^+^ CD62L^+^ T cell subset (B). ns, not significant; *P < 0.05, *n* = 5.

A further detailed analysis of the distribution of CD4^+^ AT2R^+^ T cells within CD4^+^ CD44^+^ CD62L^−^ or CD4^+^ CD44^+^ CD62L^+^ T cell subsets (Fig.4A and B) showed that a significant decrease of blood CD4^+^ AT2R^+^ T cells in patients with HF was exclusively assigned to the CD4^+^ CD44^+^ CD62L^−^ T cell subset, a known activated T cell fraction located mainly at inflammatory sites 22.

### Recruitment of CD4^+^ AT2R^+^ T cells in response to ischemic heart injury

**Figure 5:**
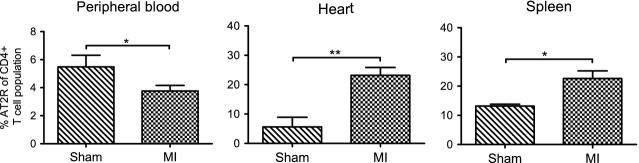
Adaptive redistribution of the CD4^+^ AT2R^+^ T cell population in response to myocardial infarction in rats. Frequency of the CD4^+^ AT2R^+^ T cells in CD4^+^ T cells of blood (sham, *n* = 7; MI, *n* = 14), heart (sham, *n* = 5; MI, *n* = 12), and spleen (sham, *n* = 3; MI, *n* = 7) was evaluated by flow cytometry analysis. Quantitative analysis reveals a significant increase of CD4^+^ AT2R^+^ T cells in post-infarct heart and spleen, coincident with a decrease in blood; *P < 0.05, **P < 0.01.

Our recently established methods 18 allowed us to elucidate a potential functional relevance of AT2R for cardiac infiltration with CD4^+^ T cell subsets. Seven days after MI, we detected CD4^+^ AT2R^+^ T cells (Fig. S2A) in infarcted myocardium, circulating blood and spleen. AT2R expression was again verified on the mRNA level. (Fig. S2B). Infiltrating CD4^+^ AT2R^+^ T cells in myocardium were mainly detected in the peri-infarct zone (Fig. S2C and Fig.5). In blood, infarcted heart and spleen, 3.8 ± 0.4%, 23.2 ± 2.7% and 22.6 ± 2.6% of CD4^+^ cells expressed AT2R, respectively (Fig.5). Together, these data reflect an adaptive and selective recruitment of the CD4^+^ AT2R^+^ T cell population into the myocardium in response to ischemic insult.

### Immunoregulatory potential of the CD4^+^ AT2R^+^ T cell population

**Figure 6:**
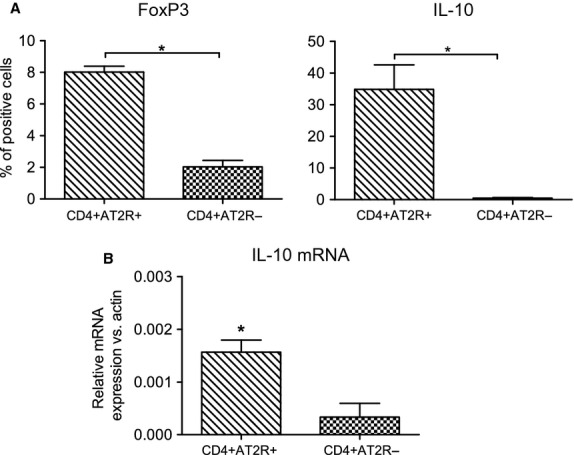
Characterization of CD4^+^ AT2R^+^ T cells in rats after myocardial infarction. FoxP3 and IL-10 (respectively, graphs on the top) were significantly up-regulated in blood CD4^+^ AT2R^+^ T cells (A) confirmed by qRT-PCR for IL-10 (B); *P < 0.05, *n* = 5.

We have shown that AT2R circumvents the cardiac inflammatory reaction *via* CD8^+^ T cells 18. To better understand the functional relevance of AT2R in CD4^+^ T cells, we dissected the expression of the immunoregulatory transcription factor FoxP3 and the cytokine IL-10 in CD4^+^ AT2R^+^ T cells derived from the peripheral blood of rats with MI. As shown in the upper panels of Figure6, the intracellular production of FoxP3 and IL-10 was significantly up-regulated 4- and 74-fold, respectively, in CD4^+^ AT2R^+^ T cells, when compared with CD4^+^ AT2R^−^ T cells (*P* < 0.05 and *P* < 0.01, respectively). These increased IL-10 expression levels in the CD4^+^ AT2R^+^ T cells were confirmed by real-time PCR analysis of *IL-10* mRNA (Fig.6, bottom). Thus, our data reveal an immunoregulatory potential of CD4^+^ AT2R^+^ T cells.

### Cardioprotective role of the CD4^+^ AT2R^+^ T cell population *in vivo*

**Figure 7:**
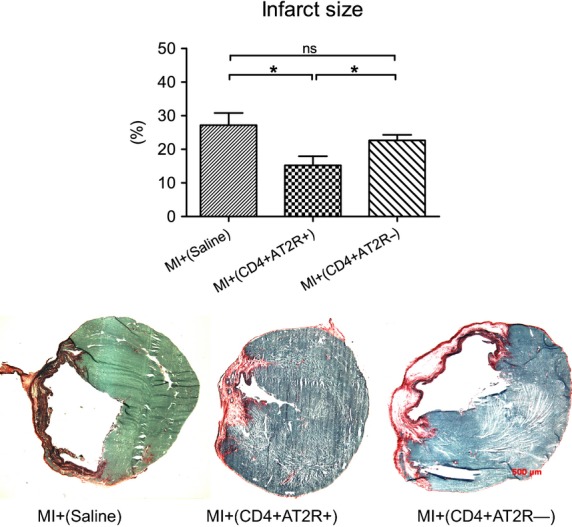
Effects of CD4^+^ AT2R^+^ T cells on cardiac injury in recipient MI rats. Intramyocardial transplantation of splenic CD4^+^ AT2R^+^ T cells (*versus* MI^+^(Saline) and CD4^+^ AT2R^−^) significantly reduced infarction size in recipient MI rats 4 weeks after transplantation. Therapy groups obtained around 2.5 × 10^5^ splenic cells, either of CD4^+^ AT2R^+^ or CD4^+^ AT2R^−^, resuspended in 50 μl saline and injected into a border zone. Control group received only 50 μl saline. Representative ventricular cross sections of infarct areas are shown in lower panels. *P < 0.05, **P < 0.01; *n* = 8; scale bar = 500 μm.

To determine whether the immunoregulatory potential of CD4^+^ AT2R^+^ T cells has functional relevance *in vivo*, we evaluated the effects of intramyocardial transplantation of splenic CD4^+^ AT2R^+^ T cells on cardiac remodelling and performance in rats with MI. Four weeks after transplantation, the engraftment of the transplanted cells from male donor rats in peri-infarct myocardium of female recipient rats was proven *via* single-colour FISH using a Y-chromosome-specific probe (Fig. S3). CD4^+^ AT2R^+^ T cells significantly ameliorated cardiac remodelling in recipient rats compared to controls. Furthermore, CD4^+^ AT2R^+^ T cells from the same donor rats, led to a significant reduction of infarction size (1.8- and 1.2- fold, respectively, Fig.7). No significant differences were observed between the control and the CD4^+^ ATR^−^ groups (*P* = 0.3171).

**Figure 8:**
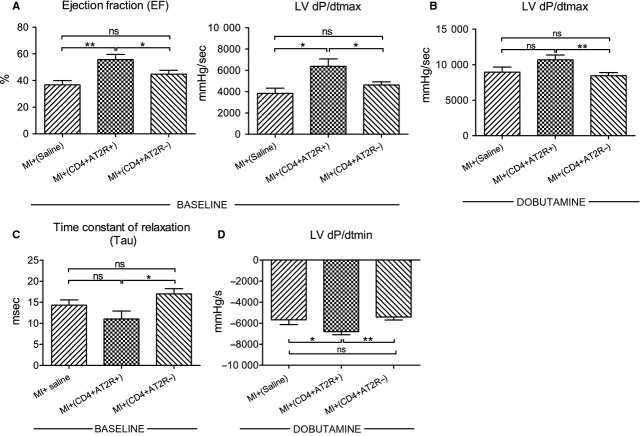
Assessment of LV functions in recipient MI rats *via* pressure-volume loops. Intramyocardial transplantation of splenic CD4^+^ AT2R^+^ T cells led to an increase in ejection fraction (EF) at steady-state and maximal peak rate of LVP (dP/dtmax, mmHg/sec.) under both baseline and stress condition (Dobutamine, 10 μg/min./kg) (A and B), and improved diastolic indices including reduced time during relaxation (Tau) at steady-state and improved minimal (−dP/dtmin., mmHg/sec.) peak rate under stress condition (C and D) as evaluated with conductance-catheter method. Around 2.5 × 10^5^ splenic cells one of the T cell subpopulation were injected in 50 μl saline into a border zone. MI^+^Saline, *n* = 10, MI^+^ CD4^+^ AT2R^+^, *n* = 10; MI^+^ CD4^+^ AT2R^−^, *n* = 15; ns: not significant, *P < 0.05, **P < 0.01.

Moreover, transplanted CD4^+^ AT2R^+^ T cells considerably improved recipient cardiac performance compared with CD4^+^ AT2R^−^ T cells and control groups. The ejection fraction at steady-state of MI plus CD4^+^ AT2R^+^ animals was increased by 1.5-fold and 1.2-fold, compared with control and MI plus CD4^+^ AT2R^−^ animals, respectively (Fig.8A and Table S2). An additional parameter relevant to cardiac systolic function, namely the maximal peak rate of LVP (dP/dtmax, mmHg/sec.) under both baseline and stress conditions as increased in CD4^+^ AT2R^+^ group. For baseline, respective values were enhanced 1.7- and 1.4-fold as compared to the control and the CD4^+^ AT2R^−^ groups (Fig.8A). Under stress, the values were elevated 1.2- and 1.3-fold in comparison to the control and the CD4^+^ AT2R^−^ groups (Fig.8B and Table S2).

Adoptive transfer of CD4^+^ AT2R^+^ T cells also markedly improved diastolic indices as shown by a reduced relaxation time at steady-state (Tau, from 17.0 ± 1.3 to 11.0 ± 1.9; Fig.8C). It likewise improved the minimal peak rate (−dP/dtmin., mmHg/sec.) under stress conditions from −6808 ± 292.2 to −5408 ± 296.0 when compared with the CD4^+^ AT2R^−^ animal group (Fig.8D and Table S2).

Overall, these data support a cardioprotective role for the CD4^+^ AT2R^+^ T cell population by ameliorating post-infarct inflammatory injury *in vivo*.

## Discussion

In this study, we report for the first time AT2R expression on CD4^+^ T cells in human as well as in rat, and describe its regulation in cardiac disease. AT2R expression on human peripheral blood CD4^+^ T cells was higher in healthy donors than in HF patients. In a rat MI model displaying cardiac remodelling with fibrosis/elevated collagen density and loss of heart function, as is typical for HF, CD4^+^ AT2R^+^ T cell number was increased in the heart and spleen but diminished in blood compared to sham-treated animals. Our findings are in accord with previous reports of elevated AT2R levels in the adult heart after MI induction 17 and specific AT2R activation after MI induction 13. Whereas previous work has mostly addressed AT2 receptor levels in whole tissues: Altarche-Xifró *et al*. showed ATR up-regulation in c-kit^+^ stem cells of rats subjected to MI, and Curato *et al*. detected splenic AT2R^+^ – expressing CD8 T cells. Based on these and our novel findings in animal models, it is likely that the mobilization of CD4^+^ AT2R^+^ T cells to the heart after RAS activation may cause their reduction in peripheral blood. Given previous observations of AT2R stimulating cell differentiation and migration 17,23, it seems likely that AT2R is actively involved in the selective extravasation of effector CD4^+^ AT2R^+^ T cells into injured myocardium.

Flow cytometric analysis of the frequency of CD4^+^ T cells in peripheral blood of patients with HF (Fig. S4) showed a significant increase compared to healthy donors, indicating the importance of this T cell subset during the progression of cardiac remodelling to HF.

It has long been appreciated that T cell subsets in inflammatory sites, like cardiac allografts and ischemic injury 24,25 down-regulate CD62L (also known as L-selectin), the lymphoid homing receptor, but up-regulate CD44, a marker of activated effector T cells 22. A reduction of CD4^+^ AT2R^+^ T cells was also found in blood CD44^+^ CD62L^−^ effector T cells of patients with HF, but not in CD44^+^ CD62L^+^ cells, confirming our hypothesis that AT2R is influenced specifically on T cell subsets involved in cardiac inflammation and regeneration.

Myocardial damage is due to a failure of self-tolerance, where infiltrating lymphocytes are reacting against self-antigens. To elucidate the role that infiltrating CD4^+^ AT2R^+^ T cells may play in this process, we analysed inflammatory properties of these cells. CD4^+^ AT2R^+^ T cells from MI rats as well as healthy and HF human donors overexpressed FoxP3 compared to CD4^+^ AT2R^−^ T cells, whereas CD25 remained unchanged. Treg cells were reported to contribute to prevention of autoimmune disease through the expression of FoxP3 26, and Tang *et al*. 27,28 observed reduced CD4^+^ CD25^+^ FoxP3^+^ CD127low Treg cells in HF, suggesting that defective Treg might be involved in disturbed immune homeostasis and also responsible for uncontrolled T cell activation in HF. While AT2R does not seem to be selective for Treg, the detected overexpression of FoxP3 suggests an anti-inflammatory potential of CD4^+^ AT2R^+^ cells.

One of the immunosuppressive properties characteristic for Treg cells is the potential to secrete IL-10 29. Notably, CD4^+^ AT2R^+^ T cells produced significantly more IL-10 than CD4^+^ AT2R^−^ T cells *in vitro*, and AngII stimulation further induced IL-10 up- as well as TNFα and interferon (IFN)-γ down-regulation (Fig. S5) in these cells. AT2R was required for the regulation, as demonstrated by selective inhibition with PD123319. The anti-inflammatory cytokine IL-10 activates JAK and/or STAT proteins leading to an induction of JAK1/Tyk2 proteins, which further activate STAT3 and SOCS3 responsible for central anti-inflammatory responses of IL-10 in macrophages. IL-10 indirectly inhibits NF-kB activation induced *via* TNF-α 30. The elevated IL-10 secretion by CD4^+^ AT2R^+^ T cells might inhibit the activation of TNF-α.

Under consideration of the above described evidence of CD4^+^ AT2R^+^ T cell anti-inflammatory potential, especially in HF individuals or under RAS stimulation, we suggested that CD4^+^ AT2R^+^ T cells of MI rats transplanted to recipient MI hearts might improve cardiac function. Indeed, pressure and volume assessment in the LVs of rats with MI plus injected splenic CD4^+^ AT2R^+^ T cells showed improved myocardial performance in comparison to those rats with CD4^+^ AT2R^−^ injected cells. Hearts derived from rats with MI^+^ (CD4^+^ AT2R^+^) presented a significant reduction in infarct size compared to hearts from the MI^+^ (CD4^+^ AT2R^−^) group. Our findings align well with studies of AT2R stimulation with Compound 21 (C21), which led to an improvement in hemodynamics and reduction in infarct size because of C21-suppressed inflammatory actions as shown by significant decrease in cytokines (IL-1β, MCP-1, IL-2, IL-6) in plasma and peri-infarct zone 13.

In an animal model of autoimmune diabetes, injected regulatory T cells were markedly present in lymph nodes rather than at the site of inflammation 31. In contrast, in a model of multiple sclerosis, transplanted cells accumulated directly at the site of inflammation in the nervous system 32. Therefore, it remains to be clarified whether the beneficial actions of transferred CD4^+^ AT2R^+^ cells observed by us occur directly in the inflamed heart or whether draining lymph nodes are involved.

Irrespective of the final site of action, we provide evidence that AT2R^+^ CD4^+^ cells possess high regenerative potential. Their selective stimulation may open up promising treatment opportunities for cardiac therapy. Here, the AT2R agonist Compound 21 may be a suitable candidate for future applications 33.

In sum, we identified CD4^+^ AT2R^+^ as a novel regulatory T cell subset with beneficial impact on cardiac function after MI. CD4^+^ AT2R^+^ T cells were up-regulated in HF patients as well as MI rats and displayed anti-inflammatory properties (overexpression of FoxP3 and IL-10, downregulation of TNF-α and IFN-γ) compared to CD4^+^ AT2R^−^ cells. Myocardial transplantation of CD4^+^ AT2R^+^ T cells led to improved cardiac function and reduced infarct size in a rat MI model. Our results introduce the AT2 receptor as a beneficial AngII-mediating receptor, implying that CD4^+^ AT2R^+^ cells are a highly promising population for regenerative therapy, as they are suitable for myocardial transplantation, pharmacological AT2R activation or a combination thereof.

## Supporting Information

**Figure S1** Identification of human blood CD4^+^ AT2R^+^ T cell population.

**Figure S2** Identification of CD4^+^ AT2R^+^ T cells in a rat MI model.

**Figure S3** Detection of male donor cells in the myocardium of female recipient rats by fluorescence *in situ* hybridization.

**Figure S4** CD4 expression in human blood mononuclear cells.

**Figure S5** Increased expression of interferon-gamma within CD4^+^ AT2R^+^ T cells of patients with HF.

**Figure S6** FACS dot plots of healthy donors and heart failure patients.
