# Supplementary material for: The CD4+AT2R+ T cell subpopulation improves post-infarction remodelling and restores cardiac function
Source: J Cell Mol Med. 2015 May 20;19(8):1975–85. doi: 10.1111/jcmm.12574 (PMC4549048; doi:10.1111/jcmm.12574)
Supplement: Supplementary file 6 [file jcmm0019-1975-sd6.pdf]

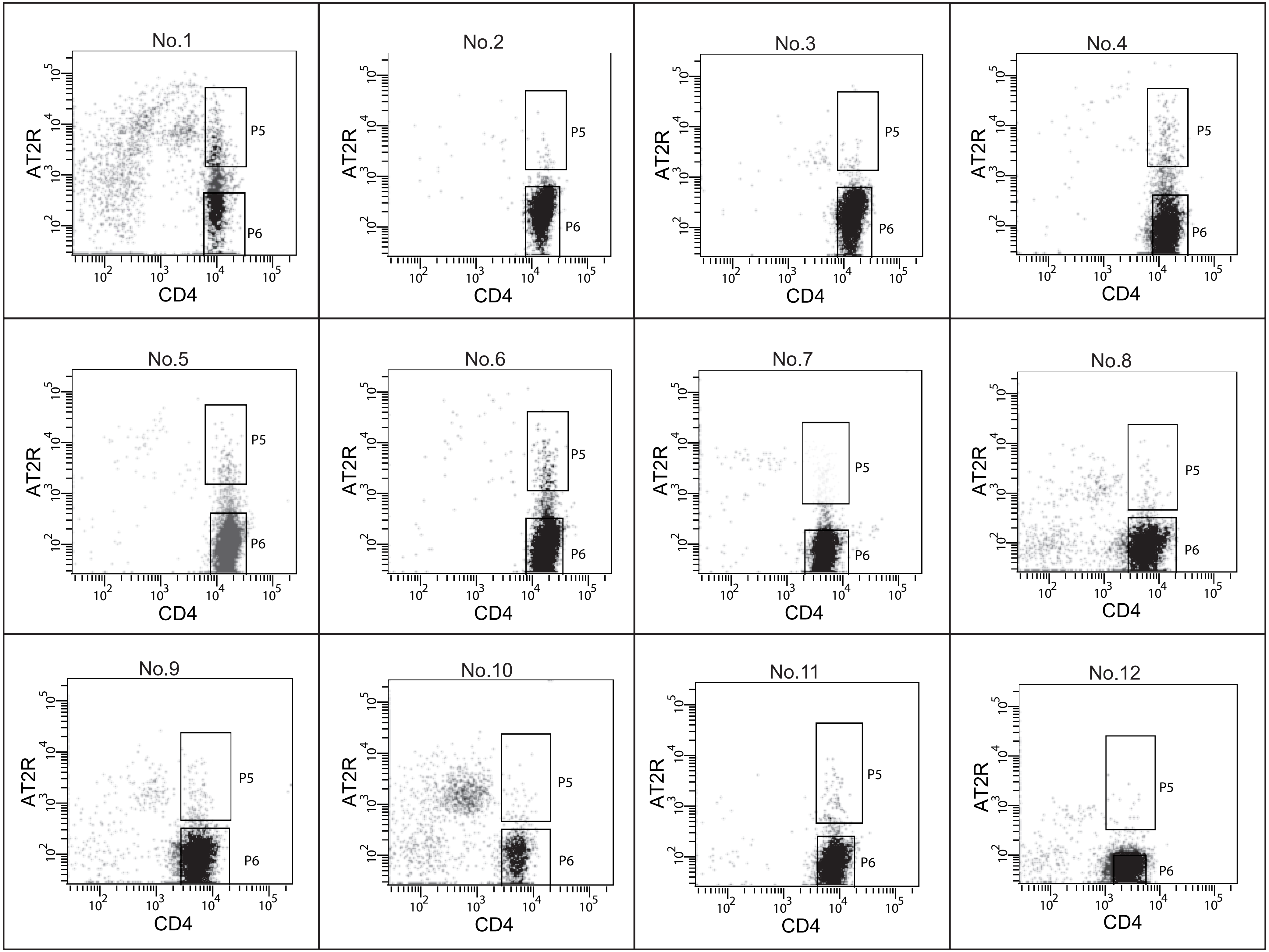
No.1
No.2
No.3
No.4
No.5
No.6
No.7
No.8
No.9
No.10
No.11
No.12
AT2R
CD4
P5
P6
10^2
10^3
10^4
10^5

Suppl.
Fig 6A

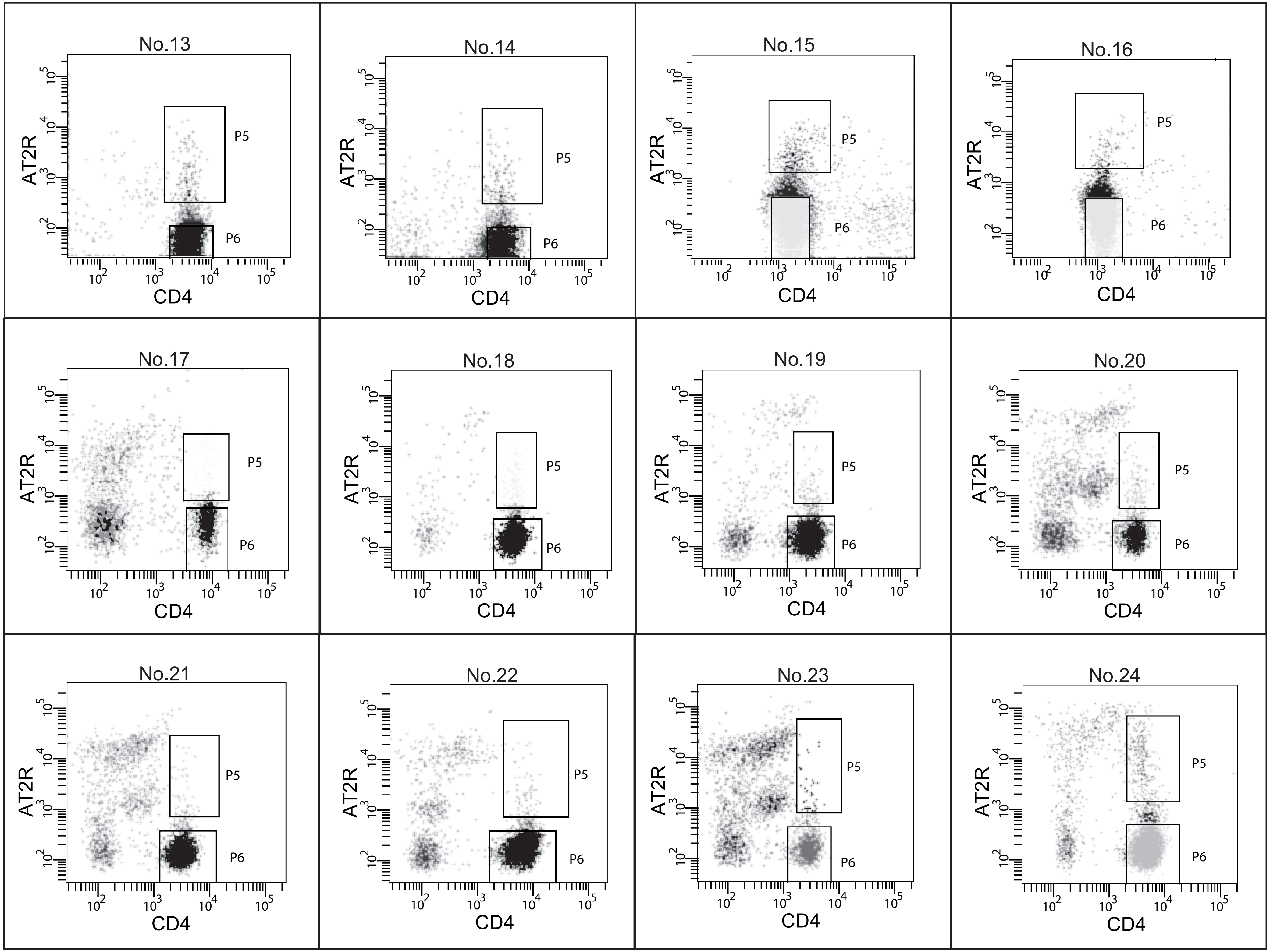
No.13
No.14
No.15
No.16
No.17
No.18
No.19
No.20
No.21
No.22
No.23
No.24
AT2R
CD4
P5
P6

Suppl. Fig 6A

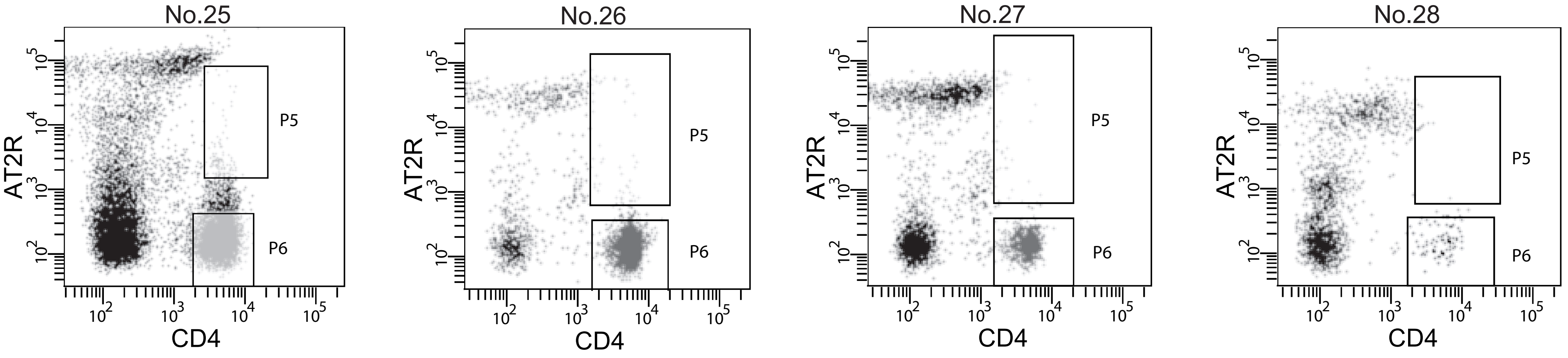

Supplementary Figure 6A

FACS dot plots of healthy donors (n=28)

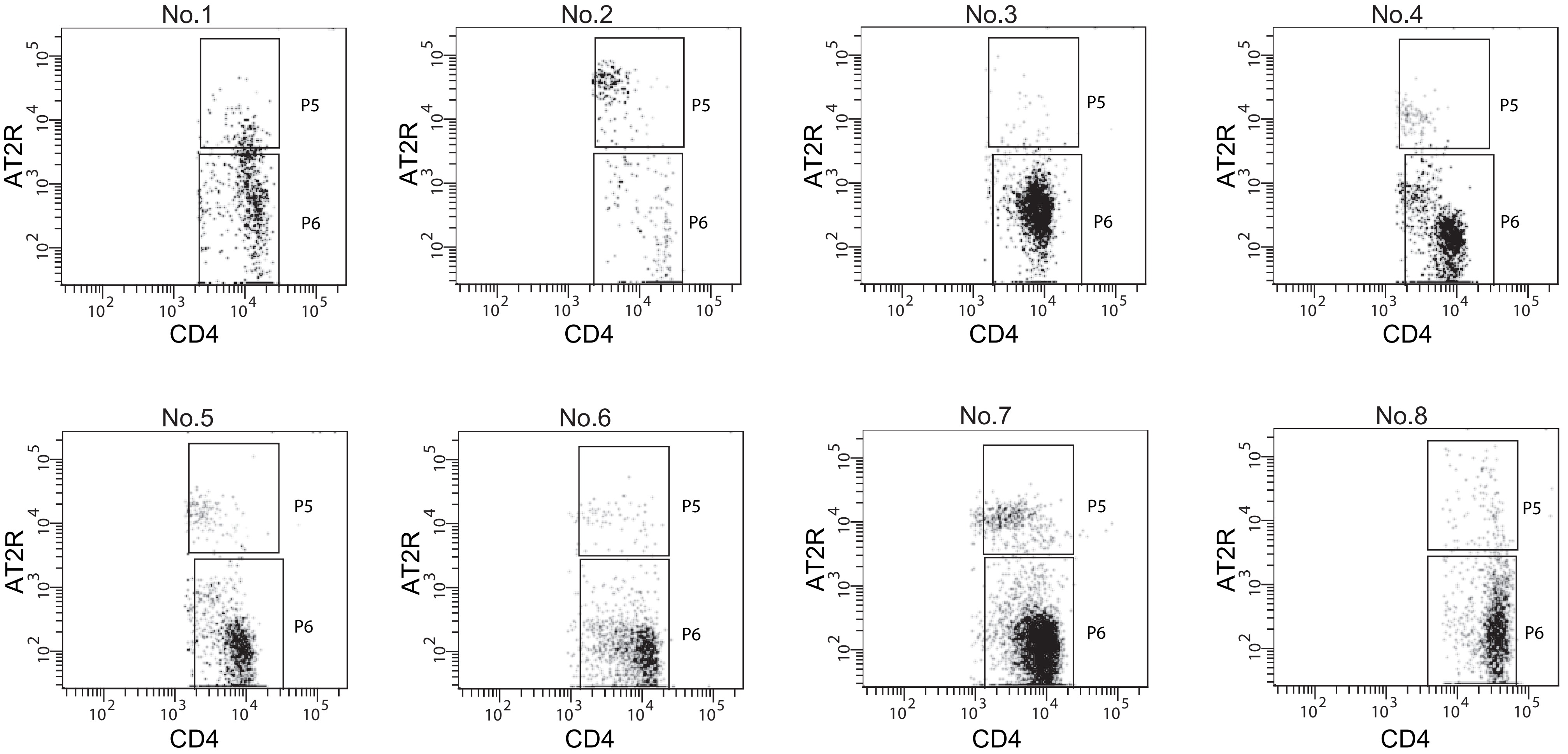

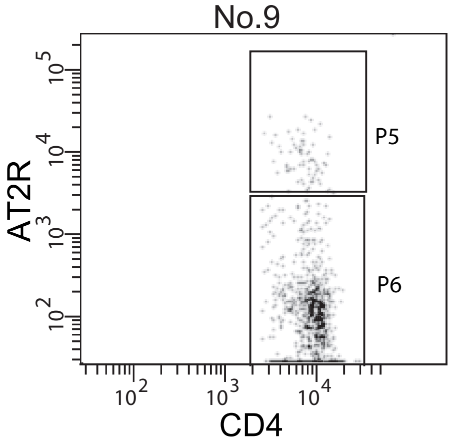

Supplementary Figure 6B

FACS dot plots of the heart failure patients (n=9)
