# Supplementary material for: The CD4+AT2R+ T cell subpopulation improves post-infarction remodelling and restores cardiac function
Source: J Cell Mol Med. 2015 May 20;19(8):1975–85. doi: 10.1111/jcmm.12574 (PMC4549048; doi:10.1111/jcmm.12574)
Supplement: Supplementary file 7 [file jcmm0019-1975-sd7.doc]

# Supporting Information

**Supplementary Figure 1. Identification of human blood CD4+AT2R+ T cell population.** **A.** Representative FACS plots of sorted CD4+AT2R+ (**1**) and CD4+AT2R- (**2**) T cells from peripheral blood of patients with heart failure (HF) and healthy donors (HD). **B.** Predominant AT2R mRNA level in sorted human blood CD4+AT2R+ (vs. CD4+AT2R-) T cells, n=3, ****p*<0.001.

**Supplementary Figure 2. Identification of CD4+AT2R+ T cells in a rat MI model** **A.** Representative FACS plots of post-infarction cardiac CD4+AT2R+ and CD4+AT2R- T cells. **B.** Accumulated AT2R mRNA levels (normalized to beta-actin) in sorted post-infarct cardiac CD4+AT2R+ (vs. CD4+AT2R-) T cells, n=3, **p*<0.05. **C.** Co-localization of AT2R in a fraction of infiltrating CD4 T cells in peri-infarct myocardium. Arrows indicate CD4+ (yellow) AT2R+ (red) cells. Sections were counterstained with DAPI (nuclei, blue). Scale bar=10µm.

**Supplementary Figure 3. Detection of male donor cells in the myocardium of female recipient rats by fluorescence *in situ* hybridization.** **A.** Four weeks after cell transplantation, donor cells were detected via fluorescence *in situ* hybridization (FISH), based on probes specific for the Y chromosome. Donor nuclei within the recipient heart are numerically labeled (nuclei *1-3*). The probes were labeled with IDYE^TM^ 556 (white) for Y chromosomes. Slides were mounted with VECTASHIELD® medium containing DAPI for counter-staining of nuclei. Z-stack images were obtained via laser scanning confocal microscopy (LSM 780, Zeiss). Y-chromosomes present within nucleus 1, 2 and 3 are also shown in *xz* and *yz* positions, respectively (Arrows in Suppl. Fig. 1**B**, 1**C** and 1**D**). Scale bar, 5 µm

**Supplementary Figure 4. CD4 expression in human blood mononuclear cells.** Mononuclear cells (MNCs) were isolated using Ficoll centrifugation method and analyzed by FACS. The frequency of CD4+ T cells is decreased in MNCs of patients with ischemic heart failure compared to healthy donors. n= 7 (HF), n=5 (control); no significance observed between both groups

**Supplementary Figure 5. Increased expression of interferon- gamma within CD4+AT2R+ T cells of patients with HF.** Mononuclear cells (MNCs) were isolated using Ficoll centrifugation method and analyzed by FACS. The frequency of IFN-γ - positive cells within the CD4+AT2R+ subset is increased in patients with ischemic heart failure compared to CD4+AT2R- T cells. n= 3, * p<0.05

**Supplementary Figure 6. FACS dot plots of healthy donors and heart failure patients.** Mononuclear cells (MNCs) were isolated from human peripheral blood using Ficoll centrifugation and stained with mouse anti-human CD4-PE and goat polyclonal anti-AT2R followed by indirect staining with secondary antibody anti-goat Alexa Fluor 488. Cells were analyzed and sorted using a BD FACS Aria II. Healthy donors, n=27; Heart failure n=9


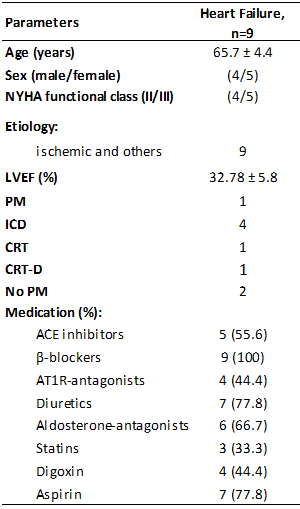


**Supplementary Table 1**. Baseline characteristics of patients with heart failure. Values are shown as mean ± SEM

**DCM: dilated cardiomyopathy, ICD: Implantable Cardioverter Defibrillator, CRT: Cardiac Resinchronization Therapy, PM: Peacemaker, AT1R: angiotensin II type 1 receptor, ACE: angiotensin-converting enzyme**

|  | **MI+(Saline), n=10** | | **MI+(CD4+AT2R+), n=10** | | **MI+(CD4+AT2R-), n=15** | | ***P* Value** | | | | | |
| --- | --- | --- | --- | --- | --- | --- | --- | --- | --- | --- | --- | --- |
|  | **Baseline** | **Dobutamine** | **Baseline** | **Dobutamine** | **Baseline** | **Dobutamine** | ***P1*** | ***P2*** | ***P3*** | ***P4*** | ***P5*** | ***P6*** |
| **Hemodynamic parameters** |  | |  |  |  |  |  | | | | | |
| Body weight (g) | 252± 46.9 | | 274.1 ± 5.902 | | 279.8 ± 5.257 | | ns | | | | | |
| HR (bmp) | 384.1 ± 16.09 | 424.8 ± 14.60 | 370.1 ± 15.38 | 434.9 ± 7.232 | 387.2 ± 9.814 | 436.1 ± 7.292 | ns | ns | ns | ns | ns | ns |
| Pmax (mmHg) | 93.17 ± 6.47 | 109.3 ± 3.912 | 105.6 ± 5.159 | 117.3 ± 3.802 | 92.08 ± 3.027 | 108.4 ± 2.341 | ns | ns | <0.05 * | ns | ns | <0.05 * |
| PES (mmHg) | 88.49 ± 7.133 | 103.3 ± 4.444 | 93.14 ± 6.692 | 104.0 ± 4.068 | 77.79 ± 2.804 | 91.23 ± 2.915 | ns | ns | <0.05 * | ns | <0.05 * | <0.05 * |
| PED (mmHg) | 9.686 ± 0.927 | 9.578 ± 0.774 | 11.66 ± 0.9256 | 10.80 ± 0.7961 | 14.25 ± 1.154 | 12.83 ± 0.9883 | ns | <0.01 ** | ns | ns | <0.05 * | ns |
| ESV (µl) | 148.7 ± 13.26 | 121.7 ± 13.49 | 143.6 ± 12.48 | 59.54 ± 6.920 | 133.1 ± 8.838 | 114.7 ± 20.09 | ns | ns | ns | <0.01** | ns | ns |
| EDV (µl) | 206.4 ± 13.13 | 201.8 ± 11.27 | 224.5 ± 13.88 | 244.4 ± 18.18 | 289.7 ± 28.82 | 241.5 ± 20.02 | ns | <0.05 * | ns | ns | ns | ns |
| SV (µl) | 73.90 ± 5.491 | 106.4 ± 13.37 | 124.5 ± 8.259 | 185.0 ± 15.86 | 152.5 ± 16.75 | 157.8 ± 15.81 | <0.01 ** | <0.01 ** | ns | <0.01 ** | <0.05 * | ns |
| CO (µl/min) | 30430 ± 3993 | 48340 ± 6979 | 54190 ± 5533 | 79890 ± 6541 | 56940 ± 5903 | 69290 ± 7054 | <0.01 ** | <0.01 ** | ns | <0.01 ** | <0.05 * | ns |
| **Systolic indices** |  | |  |  |  |  |  | | | | | |
| EF (%) | 36.80 ± 3.171 | 49.90 ± 5.448 | 55.75 ± 3.851 | 69.54 ± 4.880 | 44.74 ± 2.960 | 57.02 ± 4.081 | <0.01** | ns | <0.05 * | <0.05 * | ns | ns |
| dP/dtmax (mmHg/s) | 3828 ± 496.6 | 8946 ± 714.5 | 6370 ± 700.1 | 10690 ± 669.7 | 4620 ± 304.3 | 8444 ± 439.2 | <0.05 * | ns | <0.05 * | ns | ns | <0.01** |
| SW (µl x mmHg) | 4597 ± 661.5 | 8075 ± 1241 | 8740 ± 653.4 | 12910 ± 1676 | 7897 ± 813.0 | 13430 ± 1333 | <0.01** | <0.01 ** | ns | <0.05 * | <0.05 * | ns |
| **Diastolic indices** |  | |  |  |  |  |  | | | | | |
| -dP/dtmin (mmHg/s) | -4736 ± 747.8 | -5689 ± 447.1 | -5333 ± 570.8 | -6808 ± 292.2 | -4298 ± 293.9 | -5408 ± 296.0 | ns | ns | ns | <0.05* | ns | <0.01** |
| Tau (msec) | 14.32 ± 1.250 | 11.96 ± 0.920 | 11.03 ± 1.866 | 10.40 ± 1.482 | 16.99 ± 1.257 | 12.98 ± 0.9085 | ns | ns | <0.05 * | ns | ns | ns |

**Supplementary Table 2. Hemodynamic characteristics.** Hemodynamic characteristics measured in the left ventricular artery using pressure-volume loop (P/V-loop) method in 4-week-old rats, which undergone LAD ligation plus saline, intra-myocardial transplantation with splenic CD4+AT2R+ or CD4+AT2R- T cells. Mean ± SEM*,* ***P1:*** MI+CD4+AT2R+ vs. MI+Saline (Baseline), ***P2:*** MI+CD4+AT2R- vs. MI+Saline (Baseline), ***P3:*** MI+CD4+AT2R+ vs. MI+CD4+AT2R- (Baseline), ***P4:*** MI+CD4+AT2R+ vs. MI+CD4+AT2R- (Dobutamine), ***P5:*** MI+CD4+AT2R- vs. MI+Saline (Dobutamine), ***P6***: MI+CD4+AT2R+ vs. MI+CD4+AT2R- (Dobutamine), ns: not significant, Student T-test.

# Supplementary Methods

**Immunofluorescence staining (IF)**

Heart cryosections (5 µm) were fixed in 4% formaldehyde and incubated in Protein Block (DAKO), followed by primary goat polyclonal CD4 (1:75, Santa Cruz) and rabbit polyclonal AT2 Abs (1:50, Santa Cruz). Sections were then incubated with donkey anti-goat Alexa Fluor 647 and donkey anti-rabbit Alexa Fluor 568 (1:300, Invitrogen), and counterstained with DAPI (1:1000, Invitrogen). Stained sections were examined and analyzed under Zeiss LSM 780 Microscope.

**Quantitative RT- PCR (qRT-PCR)**

Total RNA was isolated with Trizol® Reagent (Invitrogen) or PicoPure Kit (Acturus) according to the manufacturer’s protocols. The amount of RNA was determined with NanoDrop (Thermo Scientific). 1µg total RNA was reverse transcribed to cDNA using a SuperScript II kit (Promega). Real-Time PCR (SYBR Green, Applied Biosystems) was performed for 40 cycles (95ºC 15 sec, 60ºC 1 minute, 72 °C 30 sec) using ABI 7000 and Stratagene 3000 MXP PCR cycler. The primers used (**Supplementary material Table 3, 4**) were selected to be intron spanning.

**Supplementary Table 3. Human Sybr Green Real-Time PCR primers**

| **Gene** | **Real –Time PCR primer sequence (5’ 3’)** |
| --- | --- |
| AT2R | Forward: AATATGAAGGGCAACTCCAC  Reverse: TTAAGACACAAAGGTCTCCAT |
| 18s | Forward: CCTGAGAAACGGCTACCACAT  Reverse: TTCCAATTACAGGGCCTCGA |

**Supplementary Table 4. Rat Sybr Green Real-Time PCR primers**

| **Gene** | **Real –Time PCR primer sequences (5’ 3’)** |
| --- | --- |
| AT2R | Forward: AATCCCTGGCAAGCATCTTATGT  Reverse: CGGAAATAAAATGTTGGCAATG |
|  |  |
| IL-10 | Forward: AAGGCAGTGGAGCAGGTGAA  Reverse: CGTAGGCTTCTATGCAGTTGATGA |
| β-actin | Forward: ATCGCTGACAGGATGCAGAAG  Reverse: CGCTCAGGAGGAGCAATGAT |
|  |  |

**Detection of cell engraftment by fluorescent in situ hybridization (FISH)**

Heart cryosections (5 µm) were processed for FISH to detect male donor Y chromosome. Rat IDetect™  Chromosome Y FISH Paint Probe Red was obtained from Cambio Ltd. According to the manufacturer’s protocols, sections were completely dried, rinsed with 2 x SSC at 73°C (standard saline citrate stock solution) for 2 min, treated with 0.005% pepsin working solution at 37°C for 10 min and fixed with 2.5% formaldehyde. The probe was directly placed on the sections, denatured at 69ºC for 2 min. The sealed slides were placed in a humid chamber and hybridized at 37ºC for 16 hours, Then the probe was washed off with 0.4 x SSC/0.3% Igepal® at 73ºC for 2 min and with 2 x SSC/0.1% Igepal® at room temperature for 5 min. Slides were rinsed with distilled water, dried and mounted with VECTASHIELD™ mounting medium containing DAPI (150 ng/ml, Vector Laboratories). Images were observed under Zeiss LSM 780 microscope using 63x Plan-Apochromat objective (numerical aperture (NA) = 1.4) with immersion oil (Immersol™ 518F, Carl Zeiss) at room temperature and acquired as z-stack images, consisting of 10 optical sections (1 µm each). Co-localization of Y^+^ donor cells was analyzed with Ortho module of ZEN 2010D software (Carl Zeiss).
